# 

**Published:** 1911-06

**Authors:** 


### Immortality.

In the grand and incomprehensible scheme of the limitless Universe,—instinct with Deity,—vibrant with Life and Love in every molecule of the ethereal realms of the upper deep; away, beyond the remotest world revealed to man by his ingenuity; beyond the farthest star whose light, outstripping the lightning's flash in incredible speed, requires centuries to reach our planet, there is an abode of eternal Rest and Peace. There dwells the Great First Cause, the Source and Center of that all-pervading Energy we call "Life,"—whose manifestations in myriad forms make up all the pleasing, grand and beautiful phenomena of human environment; an Intelligence, recognized by man, but whose Purpose is inscrutable. Every organism, whether animal or vegetable, is but a specialized form of that all-pervading and persistent Force. Every living thing springs from a primitive cell, and, physically, is but an aggregation of cells. In the germ of every cell there is, indwelling, the vital principle,—Life itself. These cells are not only "living," but are endowed with potentiality surpassing the ken of man, to reproduce themselves indefinitely; and with an intelligence which guides and directs them in the upbuilding of the organism; they know where to go and what to do, and do it, unerringly and well. In the acorn slumbers the giant oak. In the tiniest seed, a grand flower plant, which, germinating within its shell, bursts its bonds and, building up its stalk and stems, unfolds its leaves to kiss the sunlight and drink the dews of heaven. Under the guidance of the mysterious intelligence, in Nature's laboratory, the inorganic elements, drawn from the earth, are wrought into living tissues, and its life work is completed and crowned with a burst of bloom, whose dazzling tints please the eye—whose perfume, wafted on the breeze, intoxicates the senses and fills the air with fragrance. Thus it is with man. The soul germ is implanted by God in the tiniest embryonic cell,—an atom of protoplasm. It grows within the heart and expands with every good deed,—till at what we call "death" it bursts its earthly bindings, soars unto the Eternal, and blossoms in Immortality!

Austin, Texas. FERDINAND EUGENE DANIEL.

